# Improving diagnosis of tuberculous lymphadenitis by combination of cytomorphology and MPT64 immunostaining on cell blocks from the fine needle aspirates

**DOI:** 10.1371/journal.pone.0276064

**Published:** 2022-10-13

**Authors:** Noor Ulain, Asif Ali, Momin Khan, Zakir Ullah, Lubna Shaheen, Naveed Shareef, Muhammad Yasir, Tehmina Mustafa

**Affiliations:** 1 Department of Microbiology, Institute of Pathology and Diagnostic Medicine, Khyber Medical University, Peshawar, Pakistan; 2 Department of Histopathology, Institute of Pathology and Diagnostic Medicine, Khyber Medical University, Peshawar, Pakistan; 3 School of Medicine, University of Glasgow, Scotland, United Kingdom; 4 Department of Otolaryngology, Khyber Teaching Hospital, Peshawar, Pakistan; 5 Department of Pathology, Khyber Teaching Hospital, Peshawar, Pakistan; 6 Centre for International Health, Department of Global Public Health and Primary Care, University of Bergen, Bergen, Norway; 7 Department of Thoracic Medicine, Haukeland University Hospital, Bergen, Norway; The University of Georgia, UNITED STATES

## Abstract

**Background:**

Extra pulmonary tuberculosis (EPTB) constitutes 18% of all tuberculosis (TB) cases and tuberculous lymphadenitis (TBL) constitutes 20–40% of EPTB. Diagnosis of TBL is challenging because of the paucibacillary nature of the disease.

**Objective:**

To investigate the diagnostic potential of a new antigen detection test based on the detection of *M*. *tuberculosis* complex specific antigen MPT64 from fine needle aspirate (FNA) cytology smears and biopsies obtained from patients with clinically suspected TBL using immunohistochemistry (IHC).

**Materials and methods:**

This study was conducted at Khyber Teaching Hospital and Rehman Medical Institute, Peshawar, Pakistan, from January 2018 to April 2019. Samples, including FNA (n = 100) and biopsies (n = 8), were collected from 100 patients with presumptive TBL. Direct smears and cell blocks were prepared from the FNA samples. All samples were subjected to hematoxylin–eosin (H&E) staining, Ziehl-Neelsen (ZN) staining, and immunostaining with polyclonal anti-MPT64 antibody. The culture was performed only for biopsy specimens. All patients were followed until the completion of anti-TB treatment. The response to treatment was included in the composite reference standard (CRS) and used as the gold standard to validate the diagnostic tests.

**Results:**

The sensitivity, specificity, positive and negative predictive values for ZN staining were 4.4%,100%,100%,56%, for culture were 66%,100%,100%,50%, for cytomorphology were 100%,90.91%,90%,100%, and for immunostaining with anti-MPT64 were all 100%,respectively. The morphology and performance of immunohistochemistry were better with cell blocks than with smears.

**Conclusion:**

MPT64 antigen detection test performed better than ZN and cytomorphology in diagnosing TBL. This test applied to cell blocks from FNA is robust, simple, and relatively rapid, and improves the diagnosis of TBL.

## Introduction

Tuberculosis (TB) has claimed the lives of 1.3 million people in 2020, with an estimated 10 million new TB cases every year [1]. Extrapulmonary tuberculosis (EPTB) constitutes 18% of all TB cases according to the WHO reports, however, the proportion of EPTB is much higher among notified TB cases, from 17.4% (45,537) in 2011 to 20% (71,322) in 2016 [2]. The most common form of EPTB is the involvement of lymph nodes [3]. In low-income countries, tuberculous lymphadenitis (TBL) is the most common cause of lymphadenopathy [4, 5]. The diagnosis of TBL is challenging due to the varied clinical presentation, invasive sampling, and paucibacillary nature of the disease. The current first-line diagnostic methods in low-income settings, include hematoxylin–eosin (H&E) staining of fine needle aspiration cytology (FNAC), excisional biopsy, and Ziehl-Neelsen (ZN) staining, which have lower sensitivity and specificity and may lead to misdiagnosis. Histological features of granulomas are considered typical for tuberculosis and can also be observed in non-tuberculous mycobacterial and fungal diseases, requiring different treatment protocols. *M*. *tuberculosis* culture takes weeks; therefore, it cannot aid in timely diagnosis. Culture also requires advanced laboratory facilities with a higher bio-safety level [6]. In low-resource settings, it is common to treat patients empirically on clinical suspicion without microbiological confirmation. Incorrect diagnosis and unnecessary TB treatment have significant economic implications and lead to higher morbidity and mortality rates. Thus, there is a need for quick and reliable diagnostic tests to improve the diagnosis of TBL [7–9]. Previous studies have shown that a new antigen detection test based on the detection of *M*. *tuberculosis* complex specific antigen MPT64 from fine needle aspirate (FNA) smears and biopsies has significantly better diagnostic performance for the diagnosis of TBL than that using routine tests [4, 10–16] and is reported to be as sensitive as nested PCR [14]. However, PCR is sensitive to contamination, requires technical expertise, and is expensive for use in routine pathology laboratories. Immunohistochemistry (IHC) is a robust and relatively cost-effective technique and can easily be used in a routine histopathology laboratory compared to PCR [4, 11–18].

The aim of this study was to validate the diagnostic potential of the new MPT64 antigen detection test in a low-resource setting for the diagnosis of TBL using smears and cell blocks from FNA and biopsies obtained from patients with clinically suspected TBL.

## Materials and methods

### Study design

**Fig 1:**
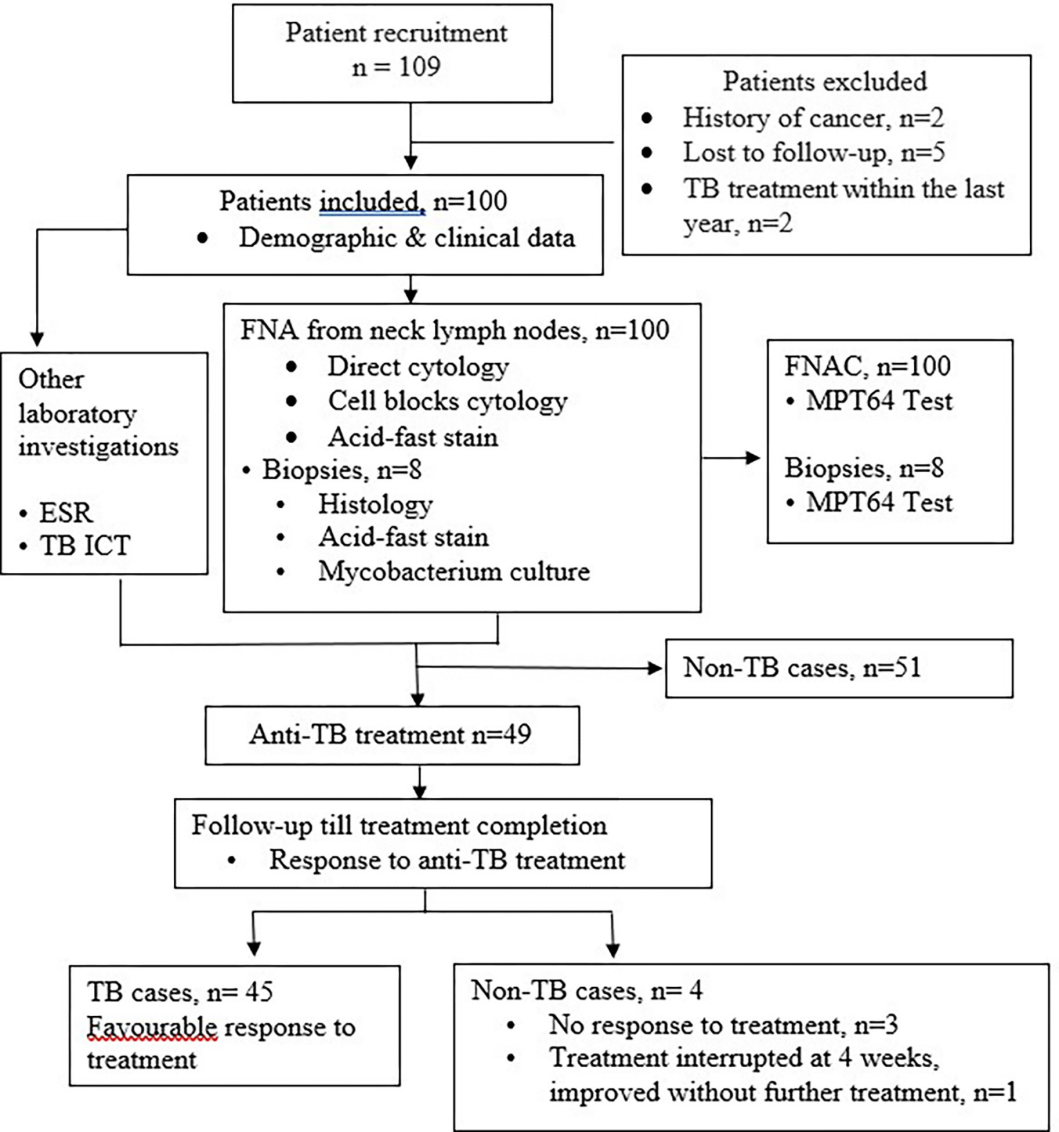
Study design, follow-up, and patient categorization into TB and non-TB cases. **Abbreviations:** EPTB, extrapulmonary tuberculosis; TB, tuberculosis; ESR, erythrocyte sedimentation rate; FNA, fine-needle aspirates; TB ICT, immunochromatographic test for tuberculosis; AFB, acid-fast bacilli.

Fig 1 shows the patient recruitment, sample collection, and patient follow-up in the study. Patients of all ages with symptoms suspected of having TBL with enlarged lymph nodes in the head and neck region attending the Otolaryngology Department, Khyber Teaching Hospital (KTH), the main tertiary care hospital in Peshawar and Rehman Medical Institute (RMI), Pakistan, from January 2018 to April 2019, were enrolled in the study. Patients with a history of cancer or those who received anti-TB treatment during the previous year were excluded from the study. Demographic and clinical data were collected on a pre-designed proforma. Samples were collected from the patients. Patients diagnosed with TBL started anti-TB treatment and were followed up for assessment of response to treatment. A favorable response to treatment was defined at 2 months based on overall clinical improvement, improvement of symptoms, regression of enlarged lymph nodes, weight gain, and decreased ESR when applicable.

Written informed consent was obtained from all patients before enrollment in the study. Ethical clearance was obtained from the Research Ethics Committee of the RMI, KTH, and Khyber Medical University.

### Preparation of FNAC samples and cell blocks

FNAC of a suspected tuberculous lymph node was performed under sterile conditions using a 23-G needle. FNAC was performed by on-duty pathologists in the pathology department, and an excisional biopsy was performed by otolaryngology surgeons of KTH and RMI in the operation theater. Half of the aspirated material was immediately smeared onto two glass slides and fixed with 95% alcohol. The fixed slides were stained by H&E and ZN stain. The other half of the FNA specimen was fixed in 4% phosphate-buffered formaldehyde for the purpose of supporting cells during transport and cell block preparation. This solution was then centrifuged for 15 min at 10,000 rpm. The supernatant fluid was removed and human blood plasma (0.25 ml) was added to the cells remaining at the bottom of the tube. A thrombin solution (0.25 ml) was added to the cell pellet. The cell pellet was left until a cell clot formed. The cell clot was then placed in formalin for 12 h and embedded in paraffin wax to make a cell block.Two consecutive, 4 μm thick sections were prepared for H&E and ZN staining. The subsequent sections were placed on special positively charged slides coated with poly-L-lysine for IHC staining.

In cases with inconclusive results, an excisional biopsy was performed. One-half of the excised lymph node was added to 4% buffered formaldehyde for the purpose of histology and IHC and the other half was added to normal saline. The specimens were decontaminated using N-acetyl-L-cysteine-sodium hydroxide before inoculation on the media. The culture was performed by inoculating the samples onto Lowenstein-Jensen egg culture medium and incubating them at 37°C for 8 weeks.

### MPT64 immunostaining

IHC was performed on formalin-fixed paraffin-embedded cell blocks and biopsies using the EnVision+System-HRP (DakoCytomation, Denmark). Briefly, sections from both the cell blocks and biopsies were deparaffinized and rehydrated in decreasing levels of alcohol.The sections were exposed to antigen retrieval in a hot-air oven using citrate buffer (pH 6.2) at 130°C for 30 min. The sections were cooled for 20 min at room temperature, and Tris-buffered saline was used for washing to remove all reagents. All sections were incubated with Dako Envision peroxidase blocking reagent for 10 min to block endogenous peroxidase reactivity. After this step, a sufficient serum-free protein block solution was applied to the sections for 10 min in a slide humidity chamber. The primary antibody was then applied at a dilution of 1:250 for 60 min, followed by incubation with the secondary antibody (Dako Envision, anti-rabbit) for 45 min in a slide humidity chamber. The antigen-antibody reaction was visualized using labeled substrate and chromogen (DAB) 3, 3’ diaminobenzidine tetrahydrochloride for 10 min and counterstained with hematoxylin for 1 min. Two groups of samples were used as negative controls, one where the full IHC protocol was followed on a known TB case without primary antibody (MPT64), and the other was the non-TB (cancer) sample. True positive or known TB samples were selected as positive controls.

### Evaluation of cytomorphology and immunostaining

The cytomorphological features compatible with TB were grouped as Group1, granulomatous inflammation without necrosis; Group 2, granulomatous inflammation with necrosis; and Group 3, only necrosis without granulomatous inflammation and without suppurative inflammation.

Cell blocks and biopsy specimens were considered positive for MPT64 immunoreactivity when there was granular brown staining within the cytoplasm of macrophages, epithelioid histiocytes, or multinucleated giant cells. The slides were studied under a light microscope using 10X which gives × magnification (100 ×). The positive signals and intensity of staining were further examined using a 40X objective which provided a magnification of 400. The mean expression of MPT64 in cytology and tissue samples was evaluated using a semi-quantitative histoscore. The staining of mycobacterial antigen MPT64 within the cytoplasm was evaluated using the histoscore formula. The histoscore included evaluation of both the staining intensity (0 = absent, 1 = weak, 2 = moderate, 3 = strong) and the proportion of positive cells. The final formula used for the calculation of histoscore was (0 × % negative cells + 1 × % weakly stained cells + 2 × % moderately stained cells + 3 × % strongly stained cells). Thus, histoscore has a possible range of 0–300. Two local pathologists assessed the slides for histoscores. The scorers were blinded to the final diagnosis. Various histoscore cutoffs were investigated for interpretation of MPT64 immunostaining using receiver operating characteristics curve (ROC) analysis. A histoscore value of 10 was used as a cut-off point to define a sample as positive for MPT64 expression based on its sensitivity and specificity of 100% respectively. There was 90% agreement between the two pathologists.

### Statistical analysis

Diagnostic validation was performed by determining the sensitivity, specificity, and positive predictive value using a 2×2 table method. For two-group comparisons, the chi-squared test was used for categorical data, and p values less than 0.05 were considered statistically significant. Data were analyzed using SPSS (version 25.0).

## Results

In total, 109 patients were enrolled in this study as shown in Fig 1. Nine patients were excluded; 5 due to loss to follow-up and 2 had received anti-TB treatment during the previous two years, and 2 had a history of cancer. Forty-nine patients were diagnosed as TB cases based on the relevant clinical features, cytology/histology findings compatible with TB, and/or positive AFB on ZN staining, and/or positive culture and were started on anti-TB treatment. Fifty-one cases that did not fulfill these criteria were diagnosed as non-TB cases. Furthermore, 3 cases did not improve on anti-TB treatment. One patient stopped treatment after 4 weeks because of side effects and improved without anti-TB treatment. These 4 cases were categorized as non-TB cases, making a total of 45 TB and 55 non-TB cases.

Table 1 shows the demographic and clinical characteristics of the patients. Compared to the non-TB group, TB patients were predominantly female (p = 0.02) and presented more frequently with symptoms. The most common symptoms were fever and weight loss. The majority of TB patients presented with cervical and multiple matted lymph nodes; 42 (93%) and 28 (62%), respectively. Positive TB history was recorded in 24 (53%) patients with TB. Relatively few TB patients presented with comorbidities; only 3(6%) patients were positive for HIV and two (4%) for diabetes.

**Table 1:** Demographic and clinical characteristics of patients.

Characteristics	TB Cases	Non-TB Cases	p-value^⁕^
	n = 45 (%)	n = 55 (%)	
**Gender**			0.027^⁕^
Male	17 (37)	34 (61)	
Female	28 (62)	21 (38)	
**Age**			0.268
≤ 14 years	5 (11)	13(23)	
15–60 years	39 (86)	41(74)	
>60	01 (2)	01(2)	
**Symptoms**			0.000^⁕^
Fever	41 (67)	20 (32)	
Weight loss	24 (53)	13(23)	
Night sweat	09(20)	02(3)	
Fever+weightloss+night sweat	41 (67)	21 (33)	
No symptoms	04(9)	34(61)	
**TB history**			0.000^⁕^
Positive	24 (53)	09(16)	
Negative	21(46)	46(83)	
**Side of lymph node**			0.005
Right	30(66)	20(36)	
Left	15(33)	35(63)	
**Number of lymph node**			0.000^⁕^
Single lymph node	17 (38)	48(87)	
Multiple matted lymphnode	28(62)	07(13)	
**Site of lymph node**			0.000^⁕^
Cervical	42(93)	29(52)	
Supraclavicular	02(4)	11(20)	
Submandibular	0(0)	14(25)	
Submental	01(2)	01(2)	
**Co-morbidities**			0.338
Diabetic	02(4)	01(2)	
HIV	03(6)	01(2)	
None	40(88)	53(96)	

^⁕^Statistically significant chi-squared test; HIV, human immunodeficiency virus; None, patients without comorbidities.

### Treatment outcome

Forty-nine patients were started on the standard anti-TB treatment. Forty-eight patients completed treatment for six months, and one patient stopped treatment after 4 weeks because of side effects and improved without anti-TB treatment. A favorable response to treatment was observed in 45 patients. Three patients did not respond to treatment.

### Cytology

**Fig 2:**
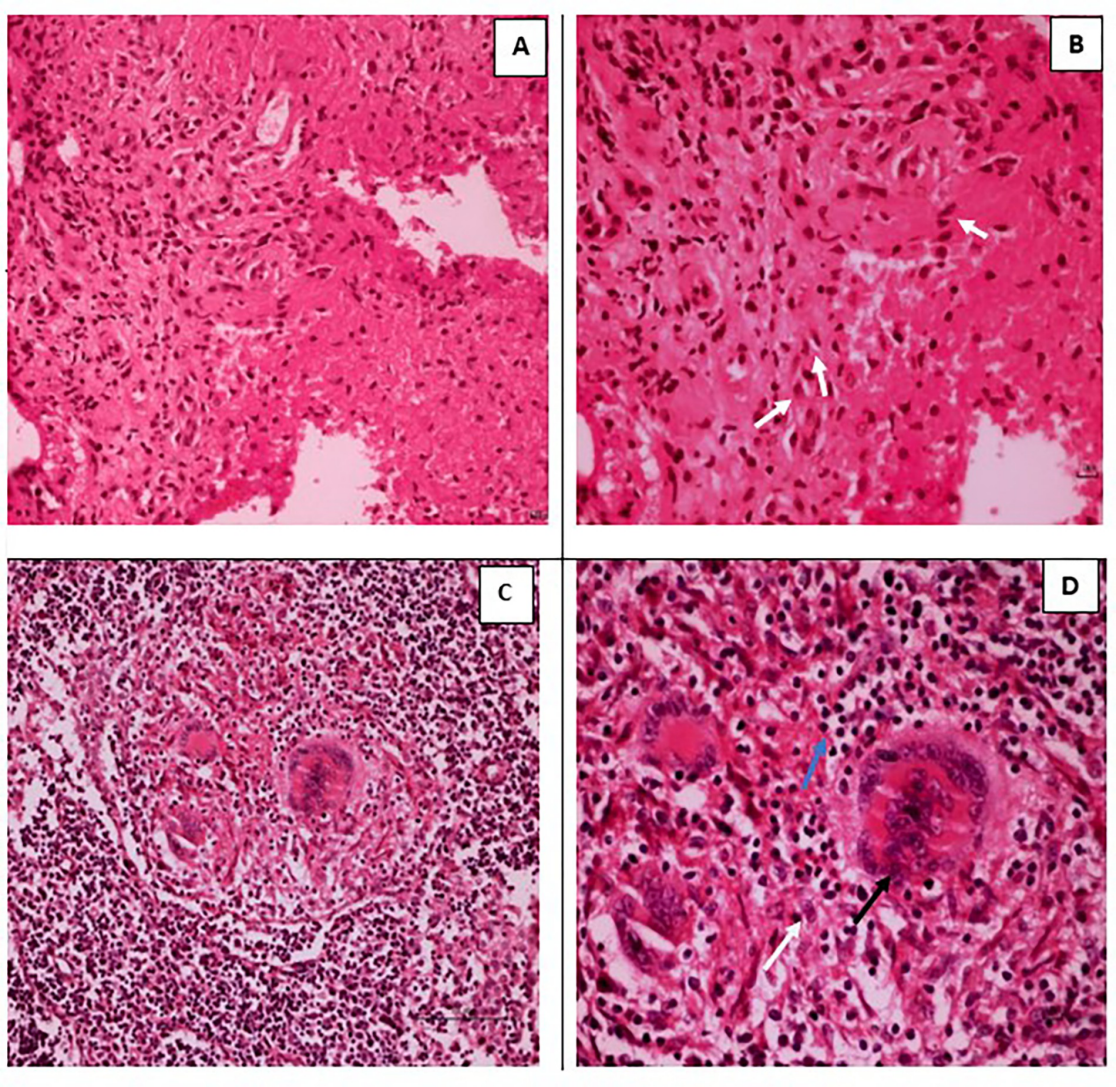
Hematoxylin & Eosin staining pattern in a tuberculous lymphadenitis case in a cell block (A,B) and a biopsy section (C,D). (A)10x, (B) 40x showing epithelioid cells (white arrow) and lymphocytes (blue arrow). (C) 10x, (D) 40x, showing epithelioid cells (white arrow), multinucleated giant cells (black arrow), and lymphocytes (blue arrow).

The patterns of cytology findings among the TB and non-TB cases are shown in Table 2. Cell blocks were available for all cases. Among the 45 TB cases, 34 (76%) and 11 (24%) showed granulomatous inflammation with (Fig 2A and 2B) and without necrosis, respectively. Among the non-TB cases, five showed features consistent with TB, one and two showed granulomatous inflammation without and with necrosis, respectively, and two showed necrosis without granulomatous and suppurative inflammation. These five cases could be atypical mycobacterial infection, multidrug-resistant TB, sarcoidosis, or fungal infections. The remaining cases showed miscellaneous pathology.

**Table 2:** The pattern of cytology findings among TB and non-TB cases.

Patterns	TB cases, n = 45	Non-TB cases, n = 55
FNAC-Cell blocks n = 100		
Granulomatous inflammation without necrosis	11	01
Granulomatous inflammation with necrosis	34	02
Necrosis without granulomatous and suppurative inflammation	NA	02
Necrosis with predominantly suppurative inflammation	NA	06
Reactive lymphoid hyperplasia	NA	27
Predominantly hemorrhage or degenerated or acellular material	NA	06
Acute inflammation	NA	03
Malignancy	NA	05
Benign tumor	NA	03
**Biopsies n = 8**	06	02
Granulomatous inflammation without necrosis	02	NA
Granulomatous inflammation with necrosis	04	NA
Reactive lymphoid hyperplasia	NA	01
Malignancy	NA	01

**Note**. FNAC, fine-needle aspiration cytology; TB, tuberculosis, NA = 0

Eight biopsy samples were studied from patients with inconclusive cytology findings. Biopsies revealed necrotic granulomas, epithelioid histiocytes, or Langhan’s giant cells in 50% of the patients, whereas 25% had non-necrotic granulomas, epithelioid histiocytes with or without multinucleated giant cells, as shown in Fig 2C and 2D. Non -TB characteristics were observed in 25% of patients.

### Immunostaining

**Fig 3:**
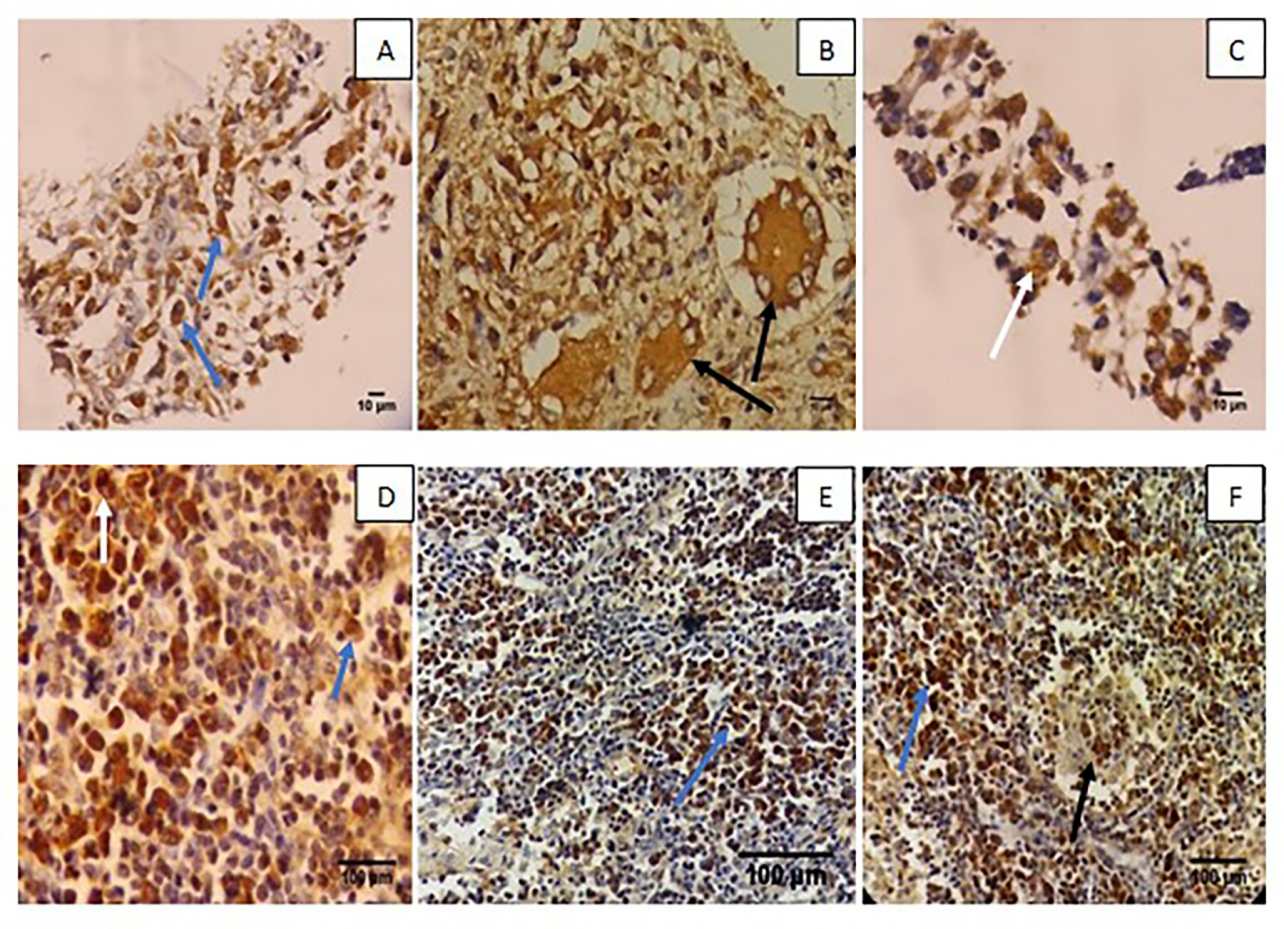
Immunohistochemical staining of cell block sections (A, B, C), and biopsy sections (D,E,F) by anti-MPT64 antibodies. A-C; Cytoplasmic staining of MPT64 in epithelioid cells (blue arrow), multinucleated giant cells (black arrow), and macrophages (white arrow). D-F; Cytoplasmic staining of MPT64 in epithelioid cells (blue arrow), macrophages (white arrow), and multinucleated giant cells (black arrow).

MPT64 immunostaining was positive in 45 TB cases and negative in 55 TB negative cases, giving an overall sensitivity and specificity of 100%, and 100%, respectively. Anti-MPT64 tended to be more frequently positive in necrotizing granulomas than in necrotic or non-necrotic granulomas. MPT64 was expressed mainly in epithelioid cells and macrophages, with or without multinucleated giant cells (Fig 3A and 3B). The staining intensity of the cells varied from weak to strong, indicating that the antigen load was present in each cell.

### Diagnostic tests among TB and non-TB cases

Table 3 shows the different diagnostic tests used for TB and non-TB cases. On the FNAC cell blocks, ZN staining was positive in 2/45 (4%) of TB blocks. Cytology consistent with TB morphology showed positive results in 45/45 (100%) TB cases and 5/55 (10%) non-TB cases. We found that MPT64 immunostaining was positive in all TB 45/45 (100%) cases and negative in 55/55 non-TB cases. Among the biopsy samples, ZN staining and culture were positive in 4/6(66%) TB cases. MPT64 was detected in all six TB cases. More antigen staining was observed in the biopsies than in the FNA cell blocks (p = 0.002).

**Table 3:** Different diagnostic tests among TB and non-TB cases.

FNAC cell blocks n = 100		TB, n = 45 (%)	Non-TB, n = 55 (%)
ZN stain	Positive	2 (4)	0
	Negative	43 (96)	55 (100)
Cytology consistent with TB	Positive	45 (100)	5 (10)
	Negative	0	50 (90)
MPT64	Positive	45 (100)	0
	Negative	0	55 (100)
MPT64 histoscore Mean (SD)		137 (34.43)^⁕^	
**Biopsies n = 8**		**TB, n = 6(%)**	**Non-TB, n = 2(%)**
ZN Stain	Positive	4 (66)	0
	Negative	2 (33)	0
Culture	Positive	4 (66)	0
	Negative	2(33)	0
Histology consistent with TB	Yes	6 (66)	0
	No	0	2 (100)
MPT64	Positive	6 (66)	0
	Negative	0	2 (100)
MPT64 histoscore Mean (SD)		223 (37.27)^⁕^	

ZN, Ziehl-Neelsen staining; FNAC, fine-needle aspiration cytology; SD, standard deviation; mean, average number; histoscore, staining intensity, and percentage of stained cells.

*Stronger staining among biopsies compared to that with FNAC (p = 0.002).

### Diagnostic validity of MPT64 immunostaining

Table 4 shows the diagnostic validation of MPT64 immunostaining and other tests in cell blocks and biopsy specimens, using CRS as a reference. Among the cell blocks, the overall sensitivity and specificity of the MPT64 antigen test were 100%, 100%; for cytomorphology were 100%, and 90%; and for ZN stain were 4.4% and 100%, respectively.

**Table 4:** Diagnostic validation of MPT64 immunostaining in cell blocks and biopsy specimens (all values are in percentage).

Diagnostic procedures	Sensitivity (95% C.I)	Specificity (95% C.I)	PPV % (95% C.I)	NPV % (95% C.I)	Accuracy % (95% C.I)
**FNACcell blocks**					
ZN stain	4.4 (0.54–15.15)	100 (93.51–100)	100	56.12 (54.57–57.67)	57 (46.71–66.86)
Cytology consistent with TB	100 (92.13–100)	90.91 (80.05–96.98)	90 (79.60–95.40)	100	95 (88.72–98.36)
MPT64 immunostaining	100 (92.13–100)	100 (92. 89–100)	100	100	100 (96.19–100)
**Biopsies**					
ZN stain	66.67 (22.28–95.67)	100 (15. 81–100)	100	50 (24.39–75.61)	75 (34.91–96. 81)
Culture	66.67 (22.2–95.67)	100 (15. 81–100)	100	50 (24.39–75.61)	75 (34.91–96. 81)
Histology consistent with TB	100 (54.07–100)	100 (15.81–100)	100	100	100 (63.06–100)
MPT64 IHC	100 (54.07–100)	100 (15. 81–100)	100	100	100 (63.06–100)

PPV, positive predictive value; NPV, negative predictive value; CI, confidence interval; ZN, Ziehl-Neelsen staining.

The MPT64 immunostaining in biopsy specimens showed sensitivity and specificity of 100% and 100%, respectively. All ZN-and culture-positive TB cases tested positive for anti-MPT64 immunostaining.

## Discussion

The MPT64 antigen detection test has been suggested as a new confirmatory test for TBL by several studies, showing its better performance compared to that of routine tests [10–16]. However, there is a need to evaluate this test in different routine diagnostic settings. In this study, we investigated the potential of the MPT64 antigen test to diagnose TBL using cell blocks made from FNAC samples at a tertiary care hospital in a resource-limited setting in the northwest province of Pakistan. We have shown that the sensitivity and specificity of the test are both 100%, indicating better performance than in other studies.

The routine clinical practice for the diagnosis of TBL at the study site is excisional biopsy with histopathological examination. However, histopathology cannot differentiate TBL from other granulomatous diseases. Furthermore, a biopsy is invasive and can exert an economic burden on patients. In contrast, FNAC of enlarged lymph nodes has been proven to be a simple, safe, quick, inexpensive, and relatively less invasive sampling technique. The preparation of cell blocks is commonly used as an adjunct to smear to enhance the diagnostic accuracy of FNAC [6, 19–22] In this study, cell blocks were prepared from FNA samples and evaluated for the diagnosis of TBL. The cytomorphological features were able to diagnose TBL with a sensitivity and specificity of 100% and 91%, respectively. The limitation of cytology is its ability to facilitate differentiation from other granulomatous conditions such as atypical mycobacterial lymphadenitis, sarcoidosis, fungal infections, brucellosis, syphilis, foreign body, and Langerhans cell histiocytosis [6], prompting the need for an add-on test to confirm TB. Immunocytochemistry was performed on the cell blocks and confirmed TB with high accuracy. Thus, the MPT64 test is an excellent adjunct to cytomorphology for the accurate diagnosis of TBL. The combination of these tests could reduce the need for more invasive excisional biopsy. The immunohistochemistry results can be obtained within 1–3 days, highlighting its utility in timely clinical decision making, reducing the diagnostic and treatment delay on one hand, and reducing unnecessary empirical treatment on the other, by excluding non-TB cases. Furthermore, the method is robust and can be performed on a bench in a pathology lab with no risk of contamination. Thus, this test can be implemented and sustained in routine pathology laboratories.

In the absence of the gold standard for the validation of a new diagnostic test in TBL, we used a composite reference standard based on the combination of clinical and laboratory findings, and response to TB treatment. However, rifampicin is a broad-spectrum antibiotic that can be used to treat many conventional bacterial infections. Therefore, the response to treatment is not conclusive for TB diagnosis and patients could have been misclassified as TB cases. We tried to account for this uncertainty by combining the cytology and the response to treatment, and all patients who responded to treatment had cytomorphological features consistent with TB. However, using CRS as the gold standard entails the risk of misclassification bias and is noted as a limitation of this study.

There is no objective biomarker for monitoring the response to treatment. Therefore, the response to treatment was assessed by the regression of enlarged lymph nodes, improvement of symptoms, and weight gain. Most of our patients showed a good response within the first two months of treatment. These findings are in agreement with other studies [23, 24], and highlight that these simple parameters can be used to monitor treatment response at a relatively early stage, and non-responders could be managed correctly and can prevent unnecessary continuation of treatment.

ZN test performed poorly for this material. It is well known that ZN staining performs poorly in TBL samples [4, 10, 11, 13, 14, 16, 25]., and a negative test does not rule out TB; thus, it does not help the clinician in decision making. This may be due to the paucibacillary nature of the disease and bacilli being below the level of detection. It can be speculated that mycobacteria may lose their acid fastness owing to the immune response within these affected areas.

We did not perform culture or Xpert MTB/RIF assays on the FNAC samples, as this was not a routine practice in our lab. This is probably due to the delay in the culture results by up to eight weeks and the need for biosafety level 3 facilities. Furthermore, culture and Xpert MTB/RIF assays have been shown to have low sensitivity for TBL sample [4, 10, 11, 13, 14, 16, 25]. However, culture remains the gold standard for TB diagnosis and is required for drug sensitivity testing. The absence of these two tests is a weakness of this study and highlights the need to strengthen the laboratory infrastructure in low-resource settings for optimal diagnosis.

The excellent performance of the MTP64 antigen detection test as compared to the routine diagnostic tests could be explained by the paucibacillary nature of the disease, and the ability of mycobacterial antigens to accumulate in the TB lesions. It has been suggested that mycobacterial antigens tend to accumulate at the site of disease and this accumulation rather than the bacillary load is the central phenomenon in TB pathogenesis and tissue destruction [26, 27].

The clinical findings of our study are in agreement with those of other studies. Cervical multiple matted lymph nodes were the most common site observed in 93% and 62% of TB cases which confirmed similar findings reported in other studies [5, 28, 29]. Our results showed that females were more frequently affected by TBL, confirming the earlier findings [5, 28, 29]. A common systemic symptom was fever (67%) associated with weight loss and night sweats, comparable to that reported in the study by Huda *et al*. [30].

Some studies have shown that *Mycobacterium bovis* is a cause of TBL instead of *Mycobacterium tuberculosis* [31]. Since MPT64 is a secreted protein and expressed by all members of the *Mycobacterium tuberculosis* complex, the MPT64 antigen test would be applicable for the diagnosis of TBL caused by *M*.*bovis*.

Unlike cell block cytology, 4/6 (66%) biopsy specimens were positive for ZN staining and cultured on LJ medium with sensitivity and specificity of 66.67% and 100%, respectively. The high sensitivity of ZN staining in biopsy specimens might be associated with a relatively larger sample volume and better sample material with biopsy than with FNAC. This may also indicate that bacteria are sequestrated in a few cells, which may be lost in the FNAC samples.

The sensitivity and specificity of cell block cytology among TB cases in our study were 100% and 91%, respectively. This result is similar to that of a study conducted to evaluate the performance of cell block, which showed a sensitivity of 100% and specificity of 96.39% in intra-abdominal mass lesions. This high sensitivity might be associated with the fact that cell blocks offer better cellular morphology than smear cytology [32]. This technique has been shown to increase the confidence level of cytopathologists in the diagnosis of lymphadenopathy [33, 34] and therefore it is recommended for routine cytomorphological analysis instead of cell smears.

## Conclusion

The MPT64 antigen detection test performed better than the ZN and cytology tests in diagnosing TBL. Cell blocks from FNA with anti-MPT64 immunostaining are robust, simple, and relatively rapid and improve the diagnosis of TBL when used with other diagnostic methods.

## Supporting information

S1 Table (XLSX)

## Abbreviations

EPTB: Extra pulmonary tuberculosis
FNAC: fine needle aspiration cytology
FNA’s: fine needle aspirates
TBL: tuberculous lymphadenitis
TB: tuberculosis
IHC: immunohistochemistry
ZN: Ziehl-Nelson stain
H&E: haematoxylin–eosin staining
AFB: acid fast bacilli
